# Tandem amplification of the *umpA* allele contributes to ceftazidime-avibactam heteroresistance in clinical carbapenem-resistant *Klebsiella pneumoniae* isolates

**DOI:** 10.1128/spectrum.01722-25

**Published:** 2026-04-13

**Authors:** Mengyao Wang, Ying Li, Jia Hu, Yu Zhang, Jing Yu, Feng Li, Yuqiao Han, Yuyuan Li, Tao Song, Yiling Lin, Yang Chen

**Affiliations:** 1 Department of Biotechnology, College of Basic Medical Science, Dalian Medical University 731355, Dalian, China; 2 Department of Clinical Laboratory, Second Affiliated Hospital of Dalian Medical University 540418, Dalian, China; 3 Advanced Institute for Medical Sciences, Dalian Medical University 36674, Dalian, China; 4 Liaoning Provincial Key Laboratory of Medical Cellular and Molecular Biology, Dalian Medical University 36674, Dalian, China; University of Guelph College of Biological Science, Guelph, Ontario, USA

**Keywords:** *Klebsiella pneumoniae*, ceftazidime-avibactam, heteroresistance, tandem amplification, *umpA*, outer membrane permeability

## Abstract

Ceftazidime-avibactam (CAZ/AVI, CZA) combination has shown promising efficacy against carbapenem-resistant *Klebsiella pneumoniae* (CRKP); however, several recent studies have reported resistance to CZA in CRKP. Hence, the present study aimed to elucidate the potential mechanisms of CZA resistance and heteroresistance in clinical carbapenemase (KPC)-producing *K. pneumoniae*. A total of 25 KPC-producing *K. pneumoniae* were collected and identified 19 CZA-resistant and 3 CZA-heteroresistant isolates using broth microdilution and population analysis profile methods, respectively. Whole-genome sequencing and comparative genomic analysis of the heteroresistant parental strain and its resistant subpopulation showed mutations in *bla*_KPC-2_ (KPC-71 variant) and tandem amplification of the lipoprotein diacylglyceryl transferase *umpA* allele in the resistant subpopulation. Reverse transcription-quantitative PCR (qRT-PCR) results demonstrated that the copy number of *umpA* gene is associated with resistance to CZA. *umpA* overexpression was induced in *K. pneumoniae* strains involved in CZA heteroresistance and failed CZA therapy in infected mice. Flow cytometry and parallel reaction monitoring (PRM) assays were performed to investigate the heteroresistance mechanisms in *umpA* overexpression strains. Flow cytometry analysis showed that overexpression of the *umpA* gene decreased propidium iodide incorporation in the cells of this strain. PRM assays showed that the peptidoglycan-associated lipoprotein Pal and the outer membrane (OM) protein OmpA were significantly upregulated in the *umpA*-overexpressing *K. pneumoniae* strain. In conclusion, we demonstrated a new mechanism for CZA heteroresistance wherein tandem *umpA* amplification generated population heterogeneity and failed CZA therapy *in vivo* experimentally infected mice. Lipoprotein biosynthetic pathways are considered a potential target for CZA resistance.

**IMPORTANCE:**

The novel synthetic β-lactamase inhibitor combination ceftazidime-avibactam has been available clinically for a few years. However, evidence that resistance and heteroresistance to ceftazidime-avibactam are responsible for treatment failure in clinical settings is increasing, and the underlying molecular mechanisms involved in developing heteroresistance have not been elucidated in detail. This study highlights that the lipoprotein diacylglyceryl transferase *umpA* gene amplification conferred heteroresistance to *K. pneumoniae* strains. Moreover, we found that *umpA* gene amplification-driven ceftazidime-avibactam heteroresistance might have resulted *in vivo* treatment failure during *K. pneumoniae* infection in mice. We also observed that the *umpA* gene amplification upregulated the expression levels of lipoproteins and outer membrane proteins and decreased outer membrane permeability in *K. pneumoniae* strains. These findings suggest that ceftazidime-avibactam heteroresistance is a major clinical concern in eradicating bacterial infections. Furthermore, lipoprotein biosynthetic pathways are considered a promising target for overcoming ceftazidime-avibactam (CZA) resistance.

## INTRODUCTION

Carbapenem-resistant Enterobacterales (CRE), including *Klebsiella pneumoniae* carbapenemase (KPC)-producing *K. pneumoniae*, constitutes a major public health concern as it increases the incidence of severe infections and complicates the therapeutic options of patients (1, 2). Recently, the cephalosporin/β-lactamase inhibitor combination ceftazidime-avibactam (CAZ/AVI, CZA) has been approved as a last-resort option for the treatment of severe infections caused by CRE producing either KPC, AmpC, and/or OXA-48-like carbapenemases, being ineffective against producers of metallo-β-lactamases (3–6).

However, since 2015, there have been increasing reports of acquired resistance to CZA in KPC-producing *K. pneumoniae* isolates (7–9). Previous studies on the mechanisms of CZA resistance have shown that most resistant isolates carried mutations in the *bla*_KPC_ gene located within the KPC enzyme omega loop, resulting in amino acid substitutions in β-lactamase and changes in gene expression (10, 11). Additionally, CZA resistance is associated with mutations in the outer membrane (OM) proteins, such as OmpK35, OmpK36, and the recently reported LamB or overexpression of efflux pumps (12, 13). However, other resistance mechanisms, particularly heteroresistance mechanisms, in clinical isolates have not yet been elucidated.

Heteroresistance, defined as the existence of a subpopulation of cells with an MIC value higher than that for the dominant population, is a frequent phenotype observed in *K. pneumoniae* isolates in the last few years (14). Heteroresistance appears to be an intermediate stage for a bacterial cell transitioning from being susceptible to being resistant after exposure to certain antibiotics, which may result in the emergence of a resistant strain and ultimately contribute to failure of antibiotic therapy. However, thus far, few studies have reported the heteroresistance phenotype and in-depth investigations on the molecular mechanisms underlying CZA heteroresistance development are lacking (15–17).

In Gram-negative bacteria, lipoproteins exhibit wide-ranging and vital biological functions such as maintenance of cell envelope architecture, insertion and stabilization of outer membrane proteins (OMPs), virulence, nutrient uptake, and transport (18). The most abundant lipoproteins in *K. pneumoniae* are the murein lipoprotein Lpp and the peptidoglycan-associated lipoprotein Pal (19). Phosphatidylglycerol:prolipoprotein diacylglyceryl transferase (Lgt) catalyzes the first step in the biogenesis of bacterial lipoproteins and plays important roles in their post-translational modifications (20). Previous functional studies on the *E. coli* Lgt coding gene *lgt* (also named *umpA,* an allele of *lgt*) have shown that Lgt depletion in a clinical strain results in OM permeabilization and increased sensitivity to serum killing and antibiotics (21). Lipoprotein biosynthetic pathways are considered an attractive target for novel antibacterial drug discovery.

Hence, the main purpose of this study was to investigate the occurrence of CZA heteroresistance in clinical CRKP strains, elucidate the potential mechanisms of heteroresistance, and evaluate the outcome of antibiotic treatment in mice infected with these heteroresistant isolates.

## RESULTS

### CZA resistance and heteroresistance in clinical CRKP isolates

The study included 25 KPC-producing CRKP strains, which exhibited resistance to at least one of three tested carbapenem antibiotics, including imipenem (IPM), meropenem (MEM), and ertapenem (ETP). Table S1 presents the CZA susceptibility, molecular and phenotypic characteristics of the strains under study (Table S1). The relative gene expression level of the *bla*_KPC_ gene between the CZA-susceptible and CZA-resistant isolates is shown in Fig. S1.

**Fig 1:**
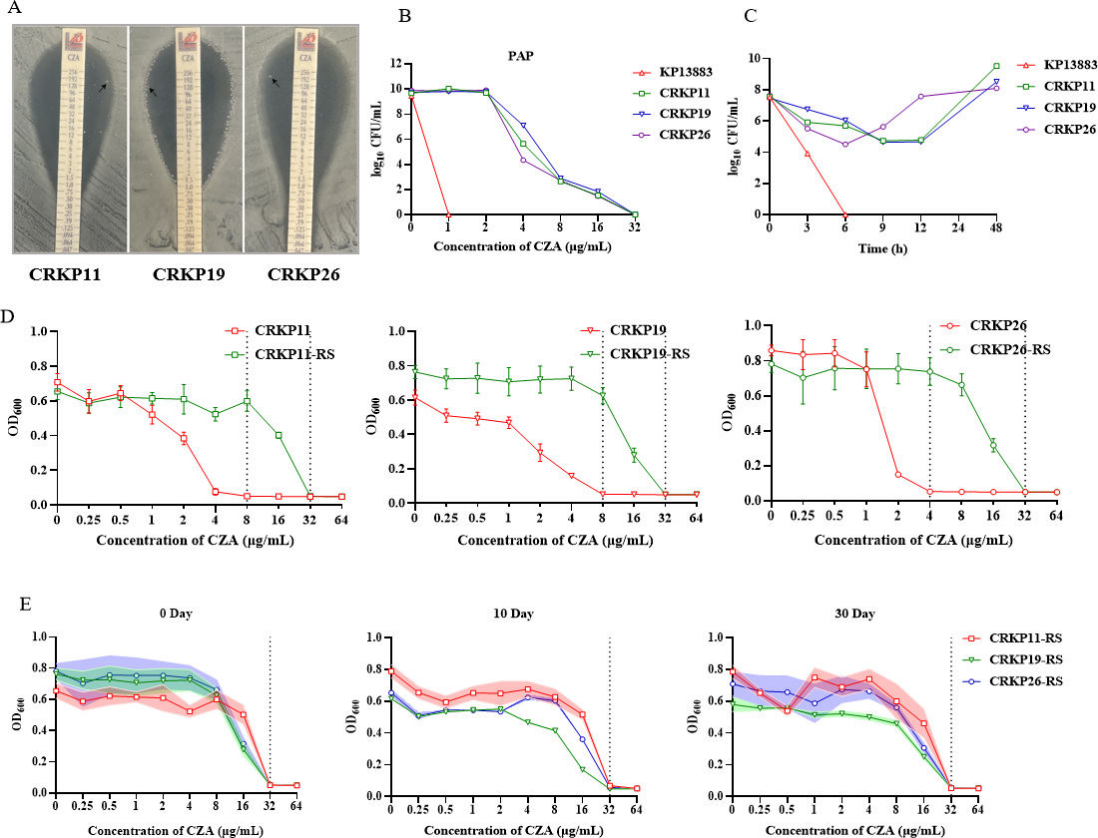
CZA-heteroresistant strains and stability of their resistant subpopulations among the 25 clinical carbapenem-resistant *Klebsiella pneumoniae* isolates. (**A**) Satellite colonies in the CZA E-test strip for the CRKP11, CRKP19, and CRKP26 strains. (**B**) Population analysis profiles of CRKP11, CRKP19, and CRKP26 and the control strain *K. pneumoniae* ATCC13883. (**C**) Time-kill kinetics assay for CRKP11, CRKP19, and CRKP26, and the control strain *K. pneumoniae* ATCC13883. (**D**) MIC values for the heteroresistant strains CRKP11, CRKP19, and CRKP26, and their resistant subpopulations (RS). Red lines indicate average values of three WT strain replicates. Green lines indicate average values of resistant subpopulation replicates. (**E**) MIC values of CZA for the passaged resistant subpopulations were determined at passages 0, 10, and 30 days. Shading indicates SEM values.

CZA heteroresistance was observed in 50% of six KPC-producing CRKP isolates with a CZA MIC value of <16/4 μg/mL (3/6; CRKP11, CRKP19, and CRKP26) (Table S1). Each heteroresistant isolate showed the growth of colonies within the inhibition zone in the CZA gradient strip testing (Fig. 1A). The resistant subpopulations of these isolates (CRKP11-RS, CRKP19-RS, and CRKP26-RS, respectively) grown on agar plates containing the highest concentrations of CZA in the PAP assay showed an MIC value of 32 µg/mL for CZA, whereas the CZA MIC values of the parental *K. pneumoniae* isolates were 4 or 8 µg/mL (Fig. 1B and D). Time-kill kinetics assay was performed to evaluate the *in vitro* antibacterial efficacy of CZA for the heteroresistant *K. pneumoniae* isolates. Although 2× MIC values of CZA exhibited a complete killing effect after 6 h for CZA-susceptible KP13883, CZA could not inhibit the heteroresistant isolates (Fig. 1C).

To examine the phenotypic stability of resistant clones from the subpopulations of the three heteroresistant isolates, the resistant subpopulations were further passaged in the absence of antibiotics and tested for changes in their resistance levels. The results showed that the three isolates maintained their resistance to CZA without any changes in their MIC values even after 30 passages (Fig. 1E; Fig. S2).

### Whole-genome sequencing (WGS) analysis

**Fig 2:**
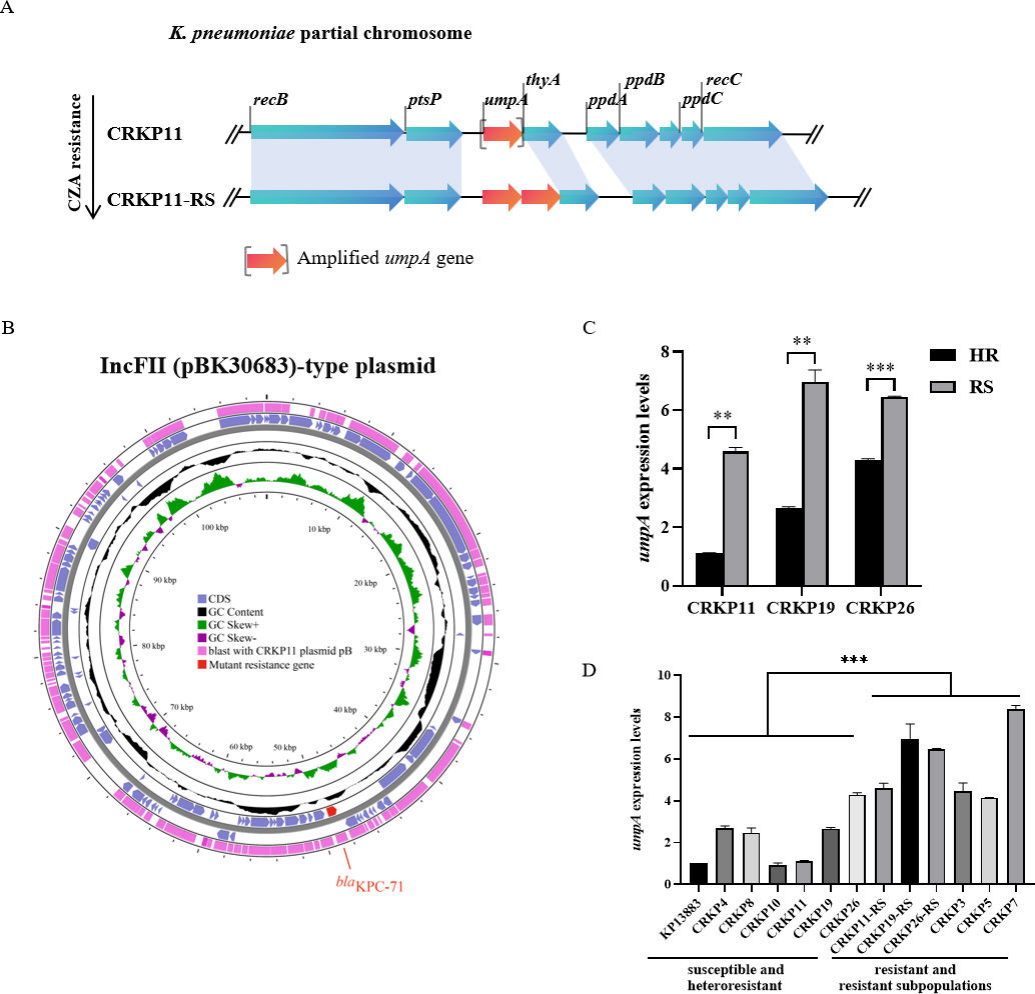
Mutations in KPCs and *umpA* tandem amplification of the chromosomal region identified by whole-genome sequencing of the heteroresistant parental strain CRKP11 and its resistant subpopulation CRKP11-RS, and qRT-PCR validation. (**A**) *umpA* gene amplification is observed in the chromosomal region of the *K. pneumoniae* CRKP11-RS strain. Red indicated the *umpA* gene. (**B**) Sequence alignment of the plasmid pB in CRKP11-RS with the sequence of the plasmid pB in CRKP11. CDS, complete coding sequence. Red regions represent the mutant KPC-71 gene. (**C**) qRT-PCR validation of the relative expression levels of the *umpA* gene in the heteroresistant strains (HR) and resistant subpopulations (RS). (**D**) The relative expression levels of the *umpA* gene in clinical KPC-CRKP isolates. All data are expressed as mean ± SEM. *n* = 4, **, *P* < 0.01, ***, *P* < 0.001 (Student’s *t*-test). *K. pneumoniae* ATCC13883 was used as the reference strain. The housekeeping gene *rrsE* was used as the endogenous reference gene.

According to the WGS data, the heteroresistant parental isolate CRKP11 and its resistant subpopulation CRKP11-RS belonged to the sequence type (ST) 15 clonal lineage. A Comprehensive Antibiotic Resistance Database (CARD)-based (http://arpcard.Mcmaster.ca, Version 1.1.3) analysis identified that CRKP11 and CRKP11-RS harbored resistance genes, including *bla*_SHV-28_, *bla*_SHV-106_, *bla*_CTX-M-15_, *aac*(6ʹ)-Ib-cr, *aac*(3)-IId, *aadA2*, *oqxA*, *oqxB*, *tet(A*), *sul1*, *fosA6*, *mph(A*), *dfrA12*, and a *bla*_KPC_ variant (data not shown). Compared with the CRKP11 isolate, the CRKP11-RS strain exhibited a tandem amplification of the chromosomal region containing the *umpA* gene (Fig. 2A).

Analysis of the genetic context of the *umpA* gene revealed that the CRKP11 and CRKP11-RS strains harbored RecBCD system, which mediates recombination through both SOS-dependent and SOS-independent mechanisms, potentially facilitating the acquisition of antibacterial resistance. In addition, the CRKP11 and CRKP11-RS strains harbored phosphorylase PtsP, thymidylate synthase thyA, and the prepilin peptidase-dependent protein genes *ppdABC* located downstream of the *umpA* gene (Fig. 2A). Furthermore, sequence analysis identified a conserved 20-bp sequence (acccgagcttccactacacg) at the junction site of the *umpA* tandem amplification. The intergenic region exhibited 100% sequence homology to the transposable elements ISC1316.

Based on plasmid sequencing, the KPC-71 variant was localized on the IncFII (pBK30683)-type plasmid (109,535 bp in length) in the CRKP11-RS strain (Fig. 2B); this variant showed a mutation of Ser182dup as compared to KPC-2. The mutant *bla*_KPC-71_ gene was flanked by IS1182 family transposase and IS481-like element ISKpn27 family transposase.

### Relationship between the copy number of the *umpA* gene and the level of CZA resistance

To analyze the copy number variation of the *umpA* gene in the CRKP11-RS, CRKP19-RS, CRKP26-RS strains as well as clinical CRKP isolates, PCR, sequencing, and qRT-PCR assays were subsequently performed. Our results indicated that *umpA* expression was significantly increased in the CZA-resistant subpopulations (CRKP11-RS: *t* = 24.665, *P* = 0.001; CRKP19-RS: *t* = 10.537, *P* = 0.008; and CRKP26-RS: *t* = 36.684, *P* < 0.001) and the clinical CZA-resistant isolates (*t* = 5.330, *P* < 0.001) as compared with that in the heteroresistant parental strains (CRKP11, CRKP19, and CRKP26) and the clinical CZA-susceptible isolates (Fig. 2C and D).

### Overexpression of *umpA* contributes to CZA heteroresistance

**Fig 3:**
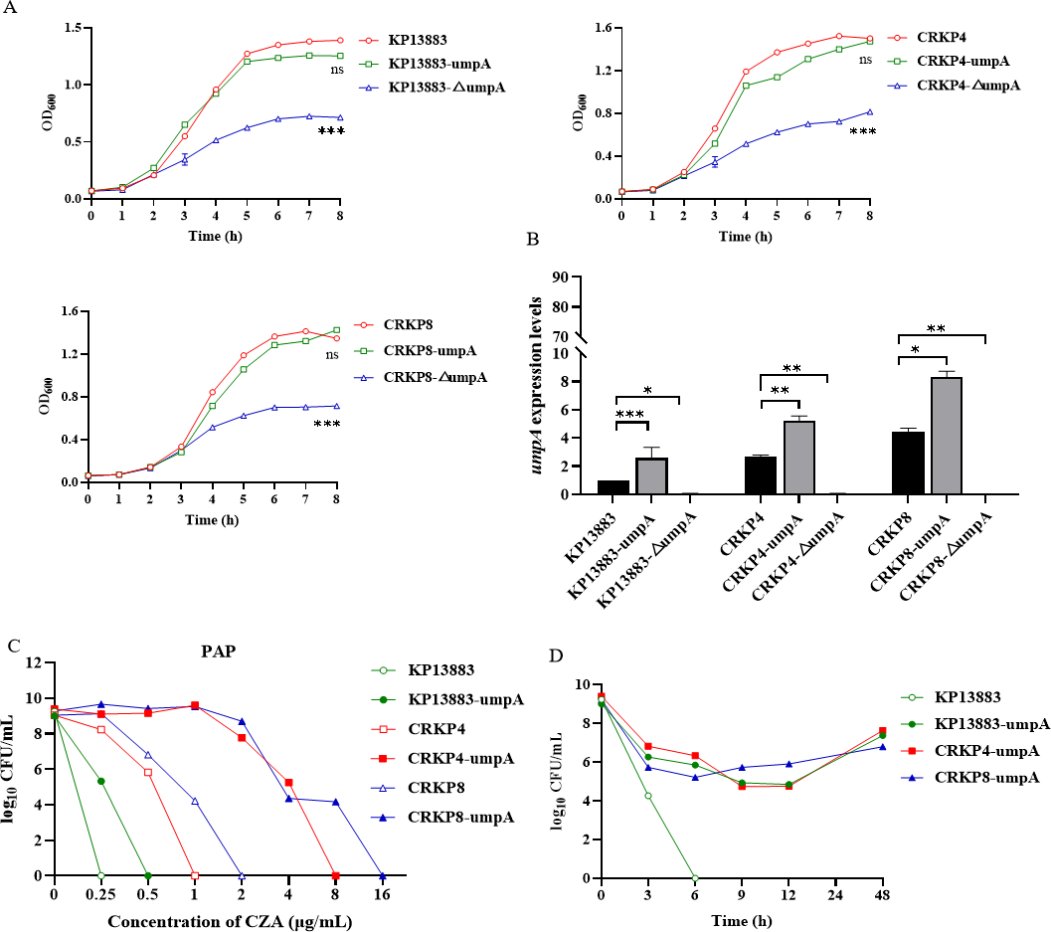
The amplification of the *umpA* gene confers heteroresistance to CZA in *K. pneumoniae* strains. (**A**) Growth curve of the *umpA* deletion and overexpression strains of *K. pneumoniae* over 8 h. *P*-values were calculated using the analysis of variance (ANOVA). (**B**) The *umpA* gene expression levels in *umpA* deletion and overexpression strains of *K. pneumoniae* were determined by qRT-PCR. All data are expressed as mean ± SEM. *n* = 4, *, *P* < 0.05, **, *P* < 0.01, ***, *P* < 0.001 (ANOVA). *K. pneumoniae* ATCC13883 was used as the reference strain. The housekeeping gene *rrsE* was used as the endogenous reference gene. (**C**) Population analysis profiles of KP13883, CRKP4, and CRKP8 strains and their *umpA* overexpression strains. (**D**) Time-kill kinetics assay for KP13883, CRKP4, and CRKP8 *umpA*-overexpressing strains and the control strain KP13883.

To confirm the hypothesis that the *umpA* gene amplification confers CZA heteroresistance to *K. pneumoniae*, we conducted two experiments involving deletion and overexpression of the *umpA* gene in KP13883, CRKP4, and CRKP8 strains. In the first experiment, the deletion mutant of *umpA* significantly decreased bacterial viability (Fig. 3A). In the second experiment, we cloned the *umpA* gene on a plasmid under the control of an arabinose-inducible promoter, which did not affect bacterial growth; the results showed that in the presence of 2% arabinose, the expression of the *umpA* gene was increased by 1.59- to 2.58-fold (Fig. 3B). As shown in Table S2, the *umpA*-overexpressing *K. pneumoniae* recombinant strains increased their CZA resistance by at least two- to fourfold as demonstrated by the MIC values ranging from 0.5 to 64 μg/mL. The three *K. pneumoniae* isolates overexpressing *umpA* (KP13883-umpA, CRKP4-umpA, and CRKP8-umpA) showed CZA heteroresistance in PAP and time-kill assays (Fig. 3C and D). This result demonstrated that the *umpA* gene overexpression led to CZA heteroresistance in clinical *K. pneumoniae* isolates.

### Therapeutic efficacy of CZA against mouse infection with *K. pneumoniae* heteroresistant strains

**Fig 4:**
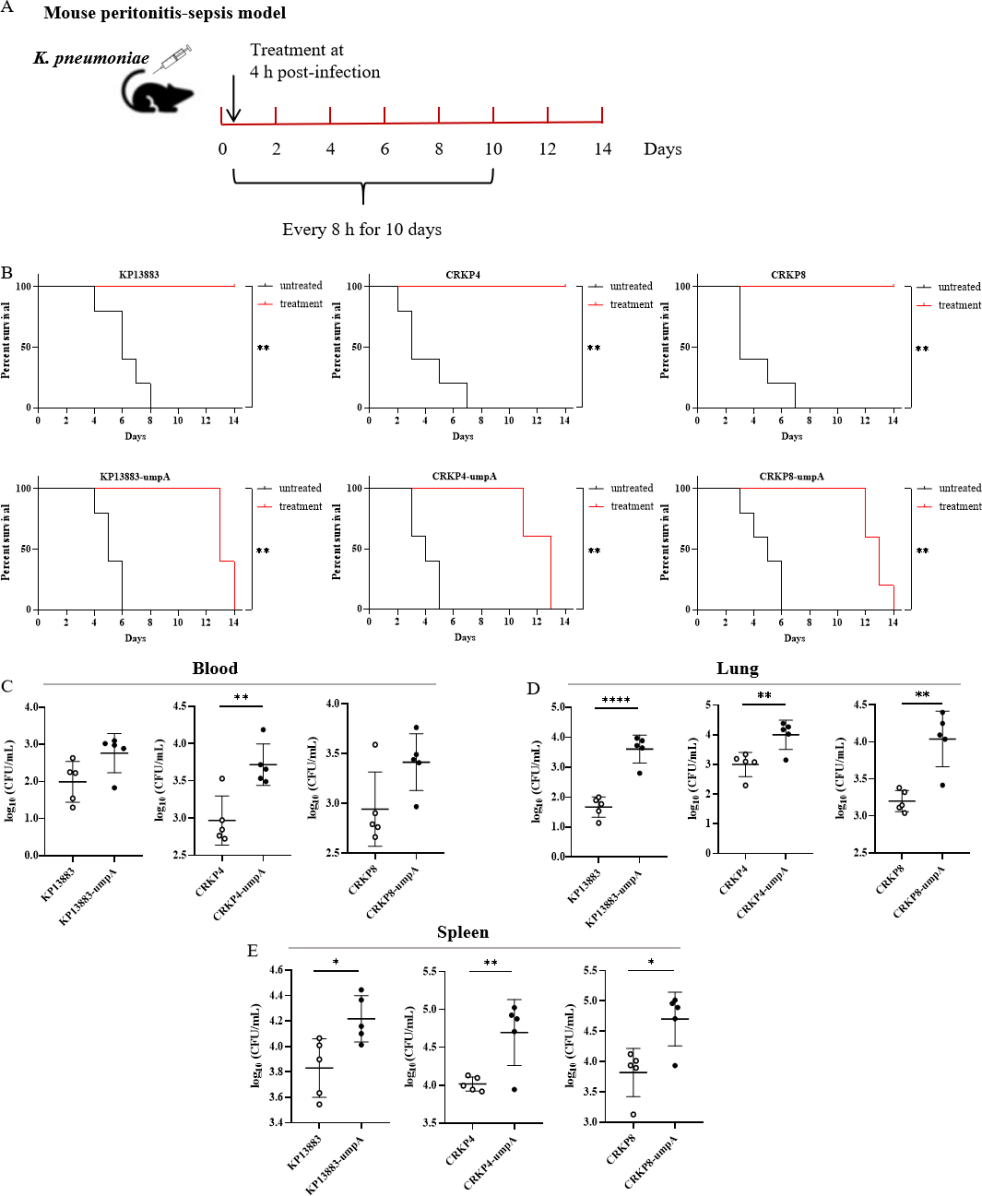
Heteroresistant *K. pneumoniae* isolates resulted in CZA treatment failure *in vivo*. (**A**) Scheme of the experimental protocols for animal infection model. (**B**) Survival of mice infected with the wild-type KP13883, CRKP4, and CRKP8 strains and their *umpA-*overexpressing strains and then treated with CZA or PBS; surviving mice were monitored until day 14 (*n* = 5). (**C–E**) Bacterial load in the blood, lung, and spleen tissues of mice at 12 days post infection (dpi). At 12 dpi, the number of viable bacterial cells in the blood, lung, and spleen tissues was determined by plating serial dilutions on agar plates (*n* = 5). Error bars represent SEM (Student’s *t*-test). *, P < 0.05; **, P < 0.01; ****, P < 0.0001.

Mice were infected with a lethal dose of either CZA-susceptible or stable CZA-heteroresistant *K. pneumoniae*; after 4 h, mice were treated with PBS or CZA for 10 days and then euthanized 2 days after treatment discontinuation. The blood, lung, and spleen tissues of mice were collected (Fig. 4A). Both susceptible and heteroresistant strains caused lethal infections in the absence of CZA within 8 days. In the CZA treatment group, mice infected with the susceptible strains (KP13883, CRKP4, and CRKP8) were rescued, whereas those infected with the heteroresistant *K. pneumoniae* strains (KP13883-umpA, CRKP4-umpA, and CRKP8-umpA) died due to infection within 4 days after the antibiotic treatment was stopped (Fig. 4B). The total bacterial load in the blood (KP13883: *t* = 2.267, *P* = 0.0531; CRKP4: *t* = 3.890, *P* = 0.0046; CRKP8: *t* = 2.248, *P* = 0.0547), lung (KP13883: *t* = 7.482, *P* <0.0001; CRKP4: *t* = 3.501, *P* = 0.0081; CRKP8: *t* = 4.660, *P* = 0.0016), and spleen (KP13883: *t* = 2.947, *P* = 0.0185; CRKP4: *t* = 3.411, *P* = 0.0092; CRKP8: *t* = 3.309, *P* = 0.0107) tissues of mice infected with susceptible strains was significantly reduced as compared with that in mice infected with the heteroresistant strains following CZA treatment (Fig. 4C through E).

### Overexpression of *umpA* results in proteomic alterations and decreases membrane permeability in *K. pneumoniae*

**Fig 5:**
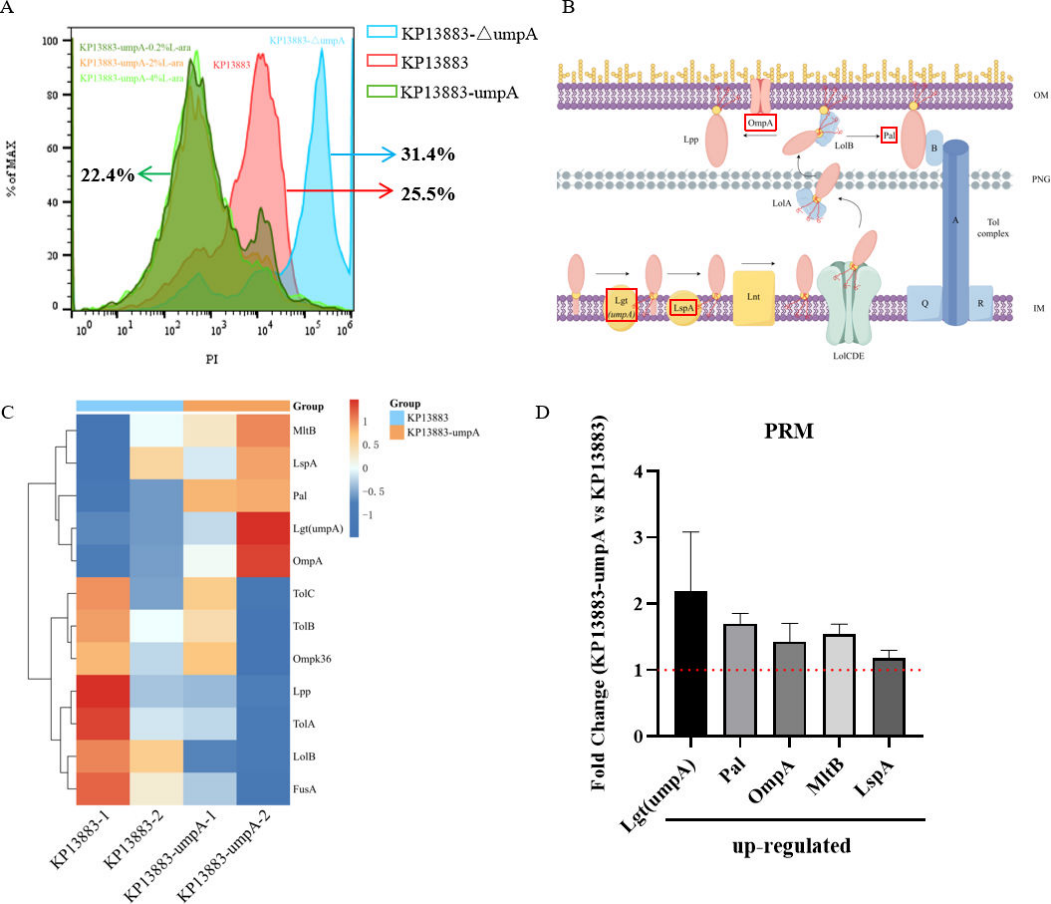
Membrane permeability and proteomic alterations in the *K. pneumoniae* ATCC13883 *umpA*-overexpressing strain. (**A**) Overexpression of the *umpA* gene decreased propidium iodide (PI) incorporation in the KP13883-umpA strain. PI is incorporated into cells with a damaged cell membrane. (**B**) Lipoprotein biosynthesis and transport in Gram-negative bacteria (obtained using Figdraw). (**C**) Heatmap of PRM mass spectrometry assay showing the 12 proteins related to bacterial lipoprotein biosynthetic pathways or membrane permeability in the KP13883 and KP13883-umpA groups. (**D**) Results of PRM analysis of upregulated proteins in the *umpA*-overexpressing *K. pneumoniae* ATCC13883 strain. Error bars represent SEM.

To determine the underlying mechanism through which *umpA* gene amplification affects heteroresistance of *K. pneumoniae* to CZA, the membrane permeability was measured based on the incorporation of PI into the damaged membranes of the cells. Deletion of the *umpA* gene displayed 31.4% PI incorporation in the cell membrane of KP13883 cells. In contrast, overexpression of the *umpA* gene, only 22.4% PI incorporation was observed, significantly reduced cell membrane integrity (*χ*^2^ = 411.9, *P* < 0.001, Fig. 5A).

The proteomic alterations were then measured in the mutant strains of the 45 proteins selected as target proteins for PRM analysis (Fig. 5B and C). As shown in Fig. 5D, we found that five proteins (Lgt [umpA], Pal, OmpA, MltB, and LspA) were upregulated in the *umpA*-overexpressing strain KP13883-umpA. According to the qRT-PCR analysis, the genes encoding the downstream enzymes in lipoprotein biosynthesis (Pal) and OM proteins (OmpA) were differentially expressed between the CZA-resistant and CZA-susceptible isolates (data not shown).

## DISCUSSION

CZA is a promising drug for treating *K. pneumoniae* infection. As shown in previous studies, patients infected with KPC-producing *K. pneumoniae* and treated with CZA exhibited increased survival as compared with those treated with other antibiotic combinations (4, 22). However, the introduction of a new drug in clinical practice resulted in the selection of beneficial mutations in endemic KPC-producing *K. pneumoniae* isolates, leading to the emergence of CZA-resistant and CZA-heteroresistant strains (23). In the present study, we found that the *umpA* gene amplification conferred CZA heteroresistance of *K. pneumoniae* strains. Furthermore, we report that the *umpA* gene amplification-driven heteroresistance may cause *in vivo* treatment failure during *K. pneumoniae* infection, which hampers the use of CZA as the last-line treatment for CRKP.

In the present study, although six clinical CRKP strains exhibited low MIC values (<16/4 μg/mL) to CZA, monitoring heteroresistance may facilitate recognition of CZA resistance development in the future. According to the results of gradient strip testing and PAP, three heteroresistant isolates were detected among the six CZA-susceptible CRKP isolates. The resistant subpopulations exhibited stable and irreversible resistance to CZA even after several passages on an antibiotic-free medium; this finding suggests that these stable resistant subpopulations may contain at least one stable mutation conferring resistance to CZA (24). Next, the WGS and analysis of the heteroresistant *K. pneumoniae* strain in the present study confirmed our assumption that the CZA-resistant subpopulation carries the KPC-2 amino acid mutation Ser182dup on the plasmid; this mutation has been identified in CZA-resistant isolates in previous studies (25).

The main finding of the present study is that *umpA* gene amplification results in the development of heteroresistance to CZA in *K. pneumoniae* strains. Tandem amplification of a gene occurs frequently in bacterial chromosomes, and previous studies have suggested that it is the most common mechanism for heteroresistance in Gram-negative bacteria (14, 26). The amplification of genes encoding efflux pumps, antibiotic targets, or antibiotic-modifying or antibiotic-degrading enzymes is involved in drug resistance development (26–28). Subsequently, we analyzed the regions surrounding the *umpA* gene. Our findings revealed that the heteroresistant strain and its resistant subpopulations harbored RecBCD, a host-encoded recombinase that plays a critical role in both initiating homologous recombination and DNA repair. The RecBCD system may facilitate the efficient acquisition of the amplified fragment (29). Analysis of intergenic sequences within the amplified *umpA* alleles identified a short-conserved sequence with substantial DNA homology to the transposable element ISC1316, suggesting that the gene amplification may be mediated by transposon activity (30).

As spontaneous tandem amplifications are intrinsically unstable and have a high cost, the mutation-causing resistance is generally unstable and lost when cells are grown in an antibiotic-deficient medium (31). However, the isolated resistant subpopulations isolated in our experiments were stable because they contained other genetic resistance mutations, such as the KPC-2 amino acid mutation, which may have only a marginal effect on the fitness of the mutants during growth in an antibiotic-deficient medium. Fitness-cost compensation could be the main factor of reversal of the resistance phenotype during cell growth in the absence of antibiotics; moreover, resistance mutations with low or no fitness cost are more likely to be stable (32).

Recent experimental and clinical studies suggest that the phenomenon of antibiotic heteroresistance might be associated with an increased risk of recurrent infections and poor treatment outcomes; this often leads to misdiagnosis and inappropriate use of antibiotics (33–36). We used a mouse peritonitis model to assess the effect of CZA therapy on stable heteroresistant *K. pneumoniae* strains with *umpA* gene overexpression. We found CZA treatment could not reduce the bacterial load in the blood, lung, and spleen tissues of mice after 12 days of infection, which resulted in CZA treatment failure when the bacterial strain exhibited a heteroresistant phenotype.

The amplified *umpA* allele observed in this study encodes the bacterial lipoprotein diacylglyceryl transferase that catalyzes the biosynthesis of lipoprotein Lpp and Pal in *K. pneumoniae*. Pal binds to the peptidoglycan layer and interacts with OmpA, Lpp, and the Tol complex and participates in maintaining cell envelope integrity. Mutation of Pal disrupts the permeability barrier of the OM and allows release of periplasmic proteins into the extracellular environment (37). Recent studies have revealed that the inhibition of the biochemical activity of Lgt interferes with the modification of the phosphatidylglycerol or prolipoprotein substrates in the OM biogenesis pathway (21); hence, further studies are required to determine which umpA-dependent lipoproteins are involved in the mechanism of CZA heteroresistance. Therefore, we conducted DDA quantitative proteomics analysis and PRM data analysis on KP13883 and *umpA*-overexpressed KP13883-umpA strains. The PRM analysis confirmed that five proteins, namely Lgt (umpA), Pal, OmpA, MltB, and LspA, which are closely associated with bacterial lipoprotein biosynthetic pathways or membrane permeability, were significantly upregulated in the *umpA*-overexpressed strain. However, the qRT-PCR assay did not reveal similar differential expression trends of *Pal* and *ompA* mRNAs between CZA-resistant and CZA-susceptible isolates (data not shown); this finding suggests that the lipoprotein diacylglyceryl transferase may modify lipoproteins at the post-transcriptional level. Flow cytometry results also confirmed reduced membrane permeability in the arabinose-induced *umpA*-overexpressing KP13883 strain. The current results indicate that the diminished cellular penetration of ceftazidime through the cell outer membrane could cause the elevated MIC value of CZA.

In conclusion, our present study revealed a new mechanism for CZA heteroresistance, wherein tandem gene amplification generated population heterogeneity. The amplification of the *umpA* gene upregulated the expression levels of Pal and OmpA and decreased OM permeability. Moreover, it induced CZA heteroresistance in clinical *K. pneumoniae* isolates and caused the failure of CZA therapy in *in vivo* experimentally infected mice. Taken together, these findings suggest that CZA heteroresistance is a major clinical concern to eradicate bacterial infections, and it is crucial to consider in CRKP therapy. Furthermore, lipoprotein biosynthetic pathways are considered a promising target for overcoming CZA resistance.

## MATERIALS AND METHODS

### Strains, antimicrobial susceptibility testing, and molecular characterization

From 2020 to 2022, 25 clinical KPC-producing CRKP isolates were obtained from different samples collected for routine microbiological testing at the university-affiliated hospitals in Dalian. The MicroScan WalkAway 96 Plus System (Siemens AG, Munich, Germany) and matrix-assisted laser desorption/ionization time-of-flight mass spectrometry (BioMérieux, Marcy l'Etoile, France) were used for bacterial identification and initial carbapenem susceptibility testing. *K. pneumoniae* isolates that were suspected to be CRKP according to the results of the MicroScan system were tested further for confirmation by the Kirby-Bauer disk diffusion method.

The MIC values of CZA were determined by the standard microdilution broth method (CZA was purchased from Solarbio, Beijing, China) or gradient strip testing (Liofilchem, Roseto degli Abruzzi, Italy), and the results were analyzed based on the clinical breakpoints recommended by the Clinical Laboratory and Standards Institute (CLSI) (38). The strains *K. pneumoniae* ATCC13883 (KP13883) and *E. coli* ATCC25922 were used for quality control.

PCR was conducted for the molecular characterization of genes (carbapenemases and ESBL-associated genes) that could explain the CZA resistance, as previously described (39). Reverse transcription-quantitative PCR (qRT-PCR) was performed to quantify the expression levels of the *bla*_KPC_ gene and the overexpression of the *umpA* gene according to the methods described in our previous study (34). The primers used for the analysis are listed in Table S3.

### Population analysis profile (PAP)

CZA heteroresistance in the *K. pneumoniae* clinical isolates was confirmed by PAP for colonies growing within the inhibitory zone in the CZA gradient strip testing (Liofilchem, Roseto degli Abruzzi, Italy). Briefly, bacterial cultures were grown overnight in Luria-Bertani (LB, Solarbio, Beijing, China) broth to the logarithmic growth phase (~10^8^  CFU/mL) and then serially diluted 10-fold with sterile saline. Next, 100 µL aliquot of each dilution was spread on freshly prepared Mueller-Hinton (MH, Solarbio, Beijing, China) agar plates containing 1 to 32 μg/mL CZA. The number of colonies was calculated after incubating for 48 h at 37°C. The experiments were repeated three times. KP13883 was used as the control strain. CZA heteroresistance was defined as the presence of CZA-susceptible isolates, among which detectable subpopulations showed growth in the presence of ≥16/4 μg/mL CZA (40). CZA-resistant subpopulations were obtained from colonies grown on MH agar plates with the highest concentrations of CZA in the PAP assay and tested in subsequent analyzes.

### Time-kill kinetics assay

Time-kill kinetics assay was performed for the CZA-heteroresistant clinical isolates. CZA was added to the broth culture in the logarithmic growth phase to yield a concentration of 16/4 μg/mL (2× MIC of the parental strains). Bacterial aliquots were collected at different time points (0, 3, 6, 9, 12, 24, and 48 h after antibiotic treatment) and inoculated into MH agar plates after appropriate dilutions. Time-kill curves were constructed by plotting mean colony counts (log10 CFU/mL) versus time*.* KP13883 was used as the reference strain.

### Stability of CZA resistance in the subpopulation

The stability of CZA resistance in the resistant subpopulation was determined, as described previously (26). Briefly, two to four clones isolated from the subpopulation with decreased susceptibility to CZA on MH agar plates were inoculated at the dilution of 1:1,000 in 4 mL of fresh LB broth for 30 passages. The MIC value of CZA for the passaged strains was determined using the standard microdilution broth method after every five passages to record any changes in the resistance pattern.

### Genomic DNA extraction and PacBio and Illumina whole-genome sequencing (WGS)

WGS was performed for the CZA-heteroresistant *K. pneumoniae* isolate (CRKP11) and its resistant subpopulation (CRKP11-RS). The detailed process of DNA extraction, library construction, and gene prediction and annotation is illustrated in Supplementary File. Briefly, single colonies of *K. pneumoniae* from an overnight agar culture plate were cultured in 200 mL of LB broth at 37°C for 12 h. Next, genomic DNA was extracted using a bacterial DNA extraction kit with magnetic beads (Majorbio, Shanghai, China) in accordance with the manufacturer’s protocol. WGS was conducted by Majorbio (Shanghai, China) with a combination of the PacBio Sequel IIe System (SMRT) and the Illumina sequencing platform to acquire complete chromosomal and plasmid sequences, respectively. Raw Illumina sequencing reads generated from the paired-end library and HiFi reads from the PacBio platform were then assembled to construct complete genomes by using Unicycle v0.4.8 (41). The coding sequences (CDSs) within the chromosomal and plasmid sequences were predicted using Glimmer or Prodigal v2.6.3 (42) and GeneMarkS (43), respectively.

### Overexpression and deletion of the *umpA* gene

Based on the *umpA* genes were included in the amplified region of CZA-resistant subpopulations, we hypothesized that CZA heteroresistance was conferred by amplification of or an increase in the copy number of the *umpA* gene. To confirm this hypothesis, we constructed *umpA* overexpression and deletion mutant strains in *K. pneumoniae* according to the method described in our previous study (44)*.* Briefly, the *umpA* gene and the homology arm sequence were amplified from KP13883 by using the primers umpA + arm-F and umpA + arm-R. The vector pBAD33 was digested with *Hind*III or *Sma*I (Takara, Dalian, China), and the PCR products were then inserted behind the arabinose (Solarbio, Beijing, China)-inducible promoter for gene overexpression. The verified overexpression plasmids were transformed into the KP13883, CRKP4, and CRKP8 strains by electroporation.

To generate *umpA* deletion mutants, the suicide vector pK18mobsacB was digested with *Hind*III and *Eco*RI (Takara, Dalian, China) and ligated with the PCR products. The transformed bacterial cells were incubated in non-kanamycin-containing LB medium with 10% sucrose and 100 μg/mL ampicillin (Solarbio, Beijing, China) at 37°C for 16 h. All primers used for the overexpression and deletion assays are listed in Table S2.

### Effect of CZA against infection caused by *umpA*-overexpressing *K. pneumoniae* strains in a mouse model

Six-week-old female C57BL/6 mice (16–18 g body weight) were obtained from the Laboratory Animal Center of Dalian Medical University (Dalian, China). Mice were housed in pathogen-free conditions with a 12-h light-dark cycle and unlimited access to food and water. All animal experimental procedures were performed in accordance with the Regulations for the Administration of Affairs Concerning Experimental Animals approved by the State Council of the People’s Republic of China. The animal experiments were conducted and approved by the Dalian Medical University Animal Care and Use Committee (Approval No. AEE18001).

Groups of 10 mice were intraperitoneally injected with 5 × 10^8^ CFU of KP13883, CRKP4, and CRKP8 wild-type strains and the heteroresistant subpopulations derived from the umpA-overexpressing strains (KP13883-umpA, CRKP4-umpA, and CRKP8-umpA, respectively) and then treated with either 1× PBS (untreated group, Solarbio, Beijing, China) or CZA (treatment group; 0.375 mg CZA/g of body weight in 0.1 mL PBS) through subcutaneous injection (45). At 12 days post-infection, five mice from each group were euthanized, and the bacterial load in their blood, lung, and spleen tissues was measured. The remaining five mice in each group were monitored for survival and weight loss until 14 days and were euthanized if their weight decreased to less than 80% of their starting weight.

### Membrane permeability assay

To determine the effect of *umpA* deletion or overexpression on bacterial membrane permeability, 1 mL culture suspensions of KP13883, KP13883-umpA, and KP13883-△umpA strains at an OD value of 0.5 were harvested, washed, and resuspended in LB broth or medium containing a range of arabinose concentrations (4%, 2%, and 0.2%), and the bacterial suspensions were incubated at 37°C for 9 h. The strains were harvested and incubated with 10 nM propidium iodide (PI, MedChemExpress, USA) in accordance with the manufacturer’s recommendations and washed with PBS (three times). The fluorescence intensity of PI was measured by flow cytometry (Mindray BriCyte E6 flow cytometer, Mindray, China), and the data were analyzed by FlowJo software.

### Protein extraction and parallel reaction monitoring (PRM) assay

Protein extraction, trypsin digestion, and quantitative proteomic data analysis were performed on KP13883 and *umpA*-overexpressing KP13883-umpA strains by Shanghai Luming Biological Technology Co., LTD (Shanghai, China). Trypsin-digested bacterial proteomic samples were analyzed on a timsTOF PRO2 mass spectrometer (Bruker Daltonics), equipped with an Acclaim PepMap C18 column (75 µm × 25 cm). Among the proteins with a significant change observed in data-dependent acquisition (DDA) mass spectrometry, 45 interesting proteins possibly associated with the bacterial lipoprotein biosynthetic pathways and membrane permeability were selected for PRM assay according to the methods described in our previous study (44). The main scanning parameters of the PASEF mode for data acquisition were as follows: capillary voltage, 1,400 V; primary and secondary scanning range of mass spectrometry, 100–1,700 m/z; and ionic mobility window range (1/k0), 0.6–1.6 vs/cm^2^. The ion accumulation and release time was set to 100 ms to achieve nearly 100% ion utilization. The time window was 10 min, and the ion migration resolution was 50. The PRM validation data were analyzed with Skyline (4.2.0, MacCoss Lab, University of Washington), and the quantification results for target peptides were manually evaluated.

### Statistical analysis

Statistical analysis was performed using SPSS software (version 17.0; SPSS) and Prism software (version 8; GraphPad). All data were obtained from at least three biological replicates and expressed as mean ± standard error of the mean (SEM). Statistical significance was determined by Student’s *t*-test between two groups, one-way factorial analysis of variance (ANOVA) among multiple groups, or nonparametric Mann-Whitney *U* tests, depending on normal/non-normal distribution of the data. Survival curves were compared using a Gehan-Breslow-Wilcoxon test. A *P*-value of <0.05 was considered statistically significant.

## Data Availability

All genome sequencing data analyzed in this study were deposited in the NCBI BioProject database under the project ID PRJNA1116829.

## SUPPLEMENTAL MATERIAL

Supplemental materials spectrum.01722-25-s0001.docx Fig. S1 and S2, Tables S1 to S3, and additional experimental details.

Graphical abstract spectrum.01722-25-s0002.tif Mechanisms of CZA heteroresistance in clinical CRKP isolates.

ASM does not own the copyrights to Supplemental Material that may be linked to, or accessed through, an article. The authors have granted ASM a non-exclusive, world-wide license to publish the Supplemental Material files. Please contact the corresponding author directly for reuse.
