# Supplementary material for: Tandem amplification of the umpA allele contributes to ceftazidime-avibactam heteroresistance in clinical carbapenem-resistant Klebsiella pneumoniae isolates
Source: Microbiol Spectr. 2026 Apr 13;14(5):e01722-25. doi: 10.1128/spectrum.01722-25 (PMC13141876; doi:10.1128/spectrum.01722-25)
Supplement: Supplemental materials — Fig. S1 and S2, Tables S1 to S3, and additional experimental details. [file spectrum.01722-25-s0001.docx]

***Supplementary file***

**Tandem amplification of *umpA* allele** **contribute to** **ceftazidime-avibactam heteroresistance in** **clinical** **carbapenem-resistant *Klebsiella pneumoniae* isolates**

*Mengyao Wang, Ying Li**, Jia Hu, Yu Zhang, Jing Yu, Feng Li,* *Yuqiao Han, Yuyuan Li, Tao Song, Yiling Lin, Yang Chen*

**Material and methods**

**DNA Extraction** Genomic DNA was extracted using Wizard® Genomic DNA Purification Kit (Promega, Madison, WI, USA) according to manufacture’s protocol. Purified genomic DNA was quantified by TBS-380 fluorometer (Turner BioSystems Inc., Sunnyvale, CA). High quality DNA (OD260/280 = 1.8-2.0, >20 μg) was used to do further research.

**Library Construction and Sequencing** Genome was sequenced using a combination of PacBio RS II Single MoleculeReal Time (SMRT) and Illumina sequencing platforms. The Illumina data was used to evaluate the complexity of the genome. For Illumina sequencing, at least 1μg genomic DNA was used for each strain in sequencing library construction. DNA samples were sheared into 400-500 bp fragments using a Covaris M220 Focused Acoustic Shearer following manufacture’s protocol. Illumina sequencing libraries were prepared from the sheared fragments using the NEXTflex™ Rapid DNA-Seq Kit. Briefly speaking, 5’ prime ends were first end-repaired and phosphorylated. Next , the 3’ ends were A-tailed and ligated to sequencing adapters. The third step is to enrich the adapters-ligated products using PCR. The prepared libraries then were used for paired-end Illumina sequencing (2 × 150 bp) onIllumina HiSeq X Ten.

For Pacific Biosciences sequencing, an aliquot of 15 μg DNA was spun in a Covaris g-TUBE (Covaris, MA) at 6,000 RPM for 60 seconds using an Eppendorf 5424 centrifuge (Eppendorf, NY). DNA fragments were then purified, end-repaired and ligated with SMRTbell sequencing adapters following manufacturer’s recommendations (Pacific Biosciences, CA). Resulting sequencing library were purified three times using 0.45 x volumes of Agencourt AMPure XP beads (Beckman

Coulter Genomics, MA) following the manufacturer’s recommendations. Next, a ~10kb insert library was prepared and sequenced on one SMRT cell using standardmethods.

**Assembly and annotation** The data generated from PacBio and Illumina platform were used for bioinformatics analysis. All of the analyses were performed using Majorbio Cloud Platform (www.majorbio.com) from Shanghai Majorbio. The detailed procedures are as follows.

**Genome Assembly** The complete genome sequence was assembled using both the PacBio reads and Illumina reads. The original image data is transferred into sequence data via base calling, which is defined as raw data or raw reads and saved as FASTQ file. Those FASTQ files are the original data provided for users, in which the read sequences and quality information are included. A statistic of quality information was applied for quality trimming, by which the low quality data can be removed to form clean data. The reads then assembled into a contig using unicycler. The last circular step was checked and finished manually, generating a complete genome with seamless chromosomes and plasmids. Finally error correction of the PacBio assembly results was performed using the Illumina reads using Pilon.

**Gene prediction and annotation** Glimmer was used for CDS prediction, tRNA-scan-SE was used for tRNA prediction and Barrnap was used for rRNA prediction. The predicted CDSs were annotated from NR, Swiss-Prot, Pfam, GO, COG and KEGG database using sequence alignment tools such as BLAST, Diamond and HMMER. Briefly, each set of query proteins were aligned with the databases, and annotations of best-matched subjects (e-value <10^-5^) were obtained for gene annotation.

**Cloning of the *bla*_KPC_ gene** Cloning experiments were performed to assess the role of *bla*_KPC_ mutations with a previously described method (1). In brief, the *bla*_KPC_ gene variant was amplified using the primers KPC+WP-F and KPC+WP-R and then cloned into the pCR-BluntII-TOPO vector (Invitrogen, Thermo Fisher, USA). Ligation mixtures pTOPO-KPC-2 and/or pTOPO-KPC-71 were introduced by transformation into the cells of chemically competent *E. coli* TOP10 (Life Technologies, Thermo Fisher Scientific, Waltham, MA, USA), *E. coli* DH5α (Takara, China), and of electrocompetent KP13883, and clinical *K. pneumoniae* isolates CRKP4 and CRKP8. Transformants were selected on LB agar plates supplemented with kanamycin (50 μg/mL) and were confirmed by sequencing of the *bla*_KPC_ gene variant.

**Data-dependent acquisition (DDA) mass spectrometry** Mass spectrometry was conducted according to the positive ionization mode and a parallel cumulative continuous fragmentation with data-dependent acquisition (DDA) approach. The capillary voltage was set at 1400 V, the primary and secondary scanning range of mass spectrometry was 100–1700 m/z, and the ionic mobility window range (1/k0) was 0.85–1.3 Vs/cm^2^. Proteins with a fold change of >1.2 and a *P*-value of <0.05 were considered as differentially expressed proteins (DEPs). The functions of DEP were analyzed according to the functional information available at the UniProt database (http://www.uniprot.org/).

**Results**

**Characterization of clinical CRKP isolates and** **CZA susceptibility** As shown in Table S1, of the 25 KPC-producing *K. pneumoniae*, all isolates carried genes encoding β-lactamases [*bla*_TEM_ (n = 22), *bla*_SHV_ (n = 25), *bla*_CTX-M-1_ (n = 5), or *bla*_CTX-M-9_ (n = 16)]. Sequencing analysis of the *bla*_KPC-2_ gene in all KPC-producing *K. pneumoniae* isolates showed that three *K. pneumoniae* isolates contained the amino acid substitution S109P located between the α3 and α4 strands of the KPC-2 enzyme. Sequencing of the OM porin genes *ompK35* and *ompK36* also showed that 6 of the 25 isolates had alterations in the L3 region of OmpK36 because of glycine and aspartic acid duplication at the amino acid residue 135 (134 to 135 GD insertion).

The relative gene expression level of the *bla*_KPC_ gene in the CZA-resistant group was 5.56 ± 1.83, which was 2.82-fold higher than that in the susceptible group (1.97 ± 0.67, *t* = 8.928, *P* <0.001, Fig. S1A and 1D). The relative gene expression level of *ompK35*/*36* showed no significant difference between the CZA-susceptible and CZA-resistant isolates (Fig. S1B-D).

**Role of *bla*_KPC-71_ mutations in high-level CZA resistance** The *E. coli* and *K. pneumoniae* recombinant reference strains carrying the KPC-71 variant showed CZA MIC values ranging from 16 to 32 μg/mL, which was 8- to 16-fold higher than that for the recombinant strain producing KPC-2. These strains also showed lower MIC values for carbapenem (Table S2). To further analyze the characteristics of the KPC-71-carrying plasmid, an electroporation experiment was performed in the CZA-susceptible *K. pneumoniae* clinical isolates CRKP4 and CRKP8; the recombinant plasmid carrying the KPC-71 gene increased the MIC value of CZA from 1 to 32 μg/mL and from 2 to 32 μg/mL in the transformants derived from the isolates CRKP4 and CRKP8 (Table S2).

**TABLE S1** Genetic and phenotypic characteristics related to ESBL, carbapenemases, and porins for the strains analyzed in this study

| Strain |  | ESBLs | |  | Carbapenemase | |  | Porins | | |  | MIC (μg/mL)  Ceftazidime/avibactam |
| --- | --- | --- | --- | --- | --- | --- | --- | --- | --- | --- | --- | --- |
|  |  | Gene | Mutation |  | Gene | Mutation |  | *ompK35* | *ompK36* | Mutation |  |  |
| CRKP3 |  | *bla*_TEM_*, bla*_SHV_*，bla*_CTX-M-9_ | W |  | KPC-2 | W |  | + | + | *ompK36* (134-135insGD) |  | 32 (R) |
| CRKP5 |  | *bla*_TEM_*，bla*_SHV_*，bla*_CTX-M-9_ | W |  | KPC-2 | W |  | + | + |  |  | 32 (R) |
| CRKP7 |  | *bla*_TEM_*，bla*_SHV_*，bla*_CTX-M-9_ | W |  | KPC-2 | W |  | + | + |  |  | 16 (R) |
| CRKP9 |  | *bla*_TEM_*，bla*_SHV_*，bla*_CTX-M-9_ | W |  | KPC-2 | W |  | + | + |  |  | 16 (R) |
| CRKP12 |  | *bla*_TEM_*，bla*_SHV_ | W |  | KPC-2 | W |  | + | + |  |  | 32 (R) |
| CRKP13 |  | *bla*_TEM_*，bla*_SHV_*，bla*_CTX-M-9_ | W |  | KPC-2 | W |  | + | + |  |  | 16 (R) |
| CRKP15 |  | *bla*_TEM_*，bla*_SHV_ | W |  | KPC-2 | W |  | + | + |  |  | 32 (R) |
| CRKP16 |  | *bla*_TEM_*，bla*_SHV_*，bla*_CTX-M-9_ | W |  | KPC-2 | W |  | + | + | *ompK36* (134-135insGD) |  | 32 (R) |
| CRKP17 |  | *bla*_TEM_*，bla*_SHV_*，bla*_CTX-M-9_ | W |  | KPC-2 | S109P |  | + | + |  |  | 32 (R) |
| CRKP18 |  | *bla*_TEM_*，bla*_SHV_*，bla*_CTX-M-9_ | W |  | KPC-2 | S109P |  | + | + | *ompK36* (134-135insGD) |  | 16 (R) |
| CRKP21 |  | *bla*_TEM_*，bla*_SHV_*，bla*_CTX-M-9_ | W |  | KPC-2 | W |  | + | + |  |  | 16 (R) |
| CRKP22 |  | *bla*_TEM_*，bla*_SHV_*，bla*_CTX-M-9_ | W |  | KPC-2 | W |  | + | + |  |  | 16 (R) |
| CRKP23 |  | *bla*_TEM_*，bla*_SHV_*，bla*_CTX-M-9_ | W |  | KPC-2 | W |  | + | + | *ompK36* (134-135insGD) |  | 32 (R) |
| CRKP24 |  | *bla*_TEM_*，bla*_SHV_*，bla*_CTX-M-9_ | W |  | KPC-2 | W |  | + | + |  |  | 32 (R) |
| CRKP25 |  | *bla*_TEM_*，bla*_SHV_*，bla*_CTX-M-9_ | W |  | KPC-2 | W |  | + | + |  |  | 16 (R) |
| CRKP27 |  | *bla*_TEM_*，bla*_SHV_*，bla*_CTX-M-9_ | W |  | KPC-2 | W |  | + | + |  |  | 64 (R) |
| CRKP28 |  | *bla*_TEM_*，bla*_SHV_ | W |  | KPC-2 | W |  | + | + |  |  | 16 (R) |
| CRKP29 |  | *bla*_TEM_*，bla*_SHV_*，bla*_CTX-M-9_ | W |  | KPC-2 | W |  | + | + | *ompK36* (134-135insGD) |  | 16 (R) |
| CRKP30 |  | *bla*_TEM_*，bla*_SHV_*，bla*_CTX-M-9_ | W |  | KPC-2 | W |  | + | + | *ompK36* (134-135insGD) |  | 16 (R) |
| CRKP11 |  | *bla*_TEM_*，bla*_SHV_*，bla*_CTX-M-1_ | W |  | KPC-2 | W |  | + | + |  |  | 8 (S)a |
| CRKP19 |  | *bla*_SHV_*，bla*_CTX-M-1_ | W |  | KPC-2 | W |  | + | + |  |  | 8 (S)a |
| CRKP26 |  | *bla*_TEM_*，bla*_SHV_*，bla*_CTX-M-1_ | W |  | KPC-2 | W |  | + | + |  |  | 4 (S)a |
| CRKP4 |  | *bla*_SHV_ | W |  | KPC-2 | W |  | + | + |  |  | 1 (S) |
| CRKP8 |  | *bla*_SHV_*，bla*_CTX-M-1_ | W |  | KPC-2 | S109P |  | + | Nd |  |  | 2 (S) |
| CRKP10 |  | *bla*_TEM_*，bla*_SHV_*，bla*_CTX-M-1_ | W |  | KPC-2 | W |  | + | + |  |  | 2 (S) |

*^a^* The strains were heteroresistance to ceftazidime-avibactam.

Abbreviations: ESBL, extended-spectrum β-lactamases. W, wild-type; ins, insertion; Nd, Not determined.


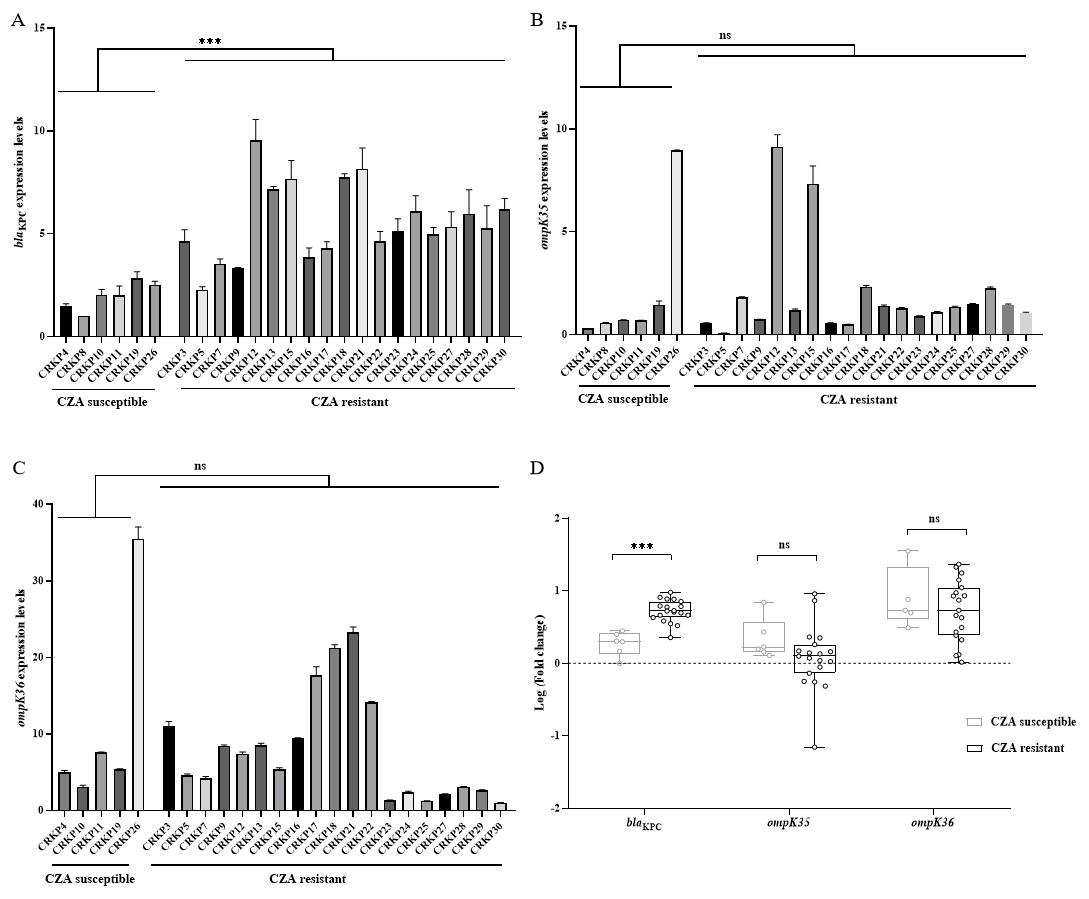


**FIG S1** The relative expression levels of genes in the 25 KPC-producing *Klebsiella pneumoniae* isolates. (A) *bla*_KPC_. (B) *ompK35*. (C) *ompK36*. (D) Expression fold change between ceftazidime-avibactam-resistant (CZA-resistant) and ceftazidime-avibactam-susceptible (CZA-susceptible) isolates. All data are expressed as mean ± SEM. n = 4, ***, *P* <0.001; ns, not significant (Student’s *t*-test). *K. pneumoniae* ATCC13883 was used as the reference strain. The housekeeping gene *rrsE* was used as the endogenous reference gene.


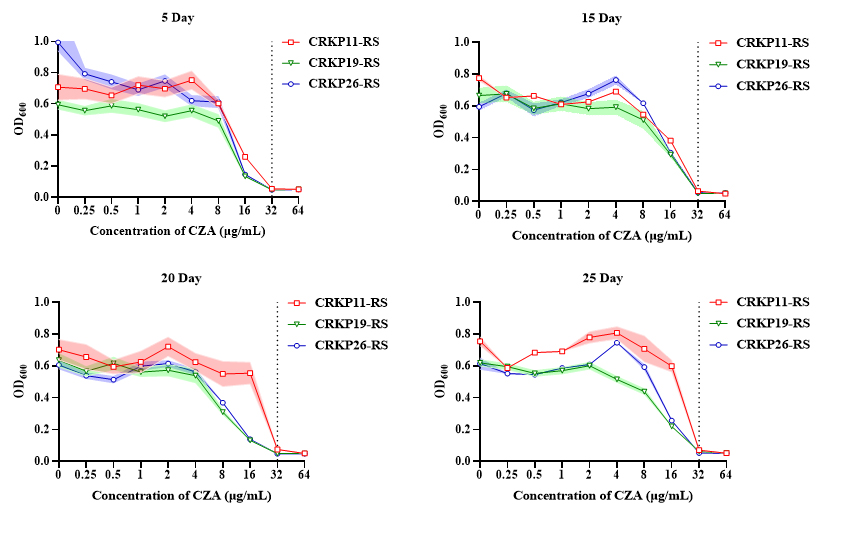


**FIG S2** MIC values of CZA for the passaged resistant subpopulations CRKP11-RS, CRKP19-RS, and CRKP26-RS, were determined at passages 5, 15, 20, and 25 days. Shading indicates SEM values.

| TABLE S2 Susceptibility of *K. pneumoniae* and *E. coli* recombinants, transformants, and recipient strains to antimicrobial agents*^a^* | | | | | | | | |
| --- | --- | --- | --- | --- | --- | --- | --- | --- |
|  |  |  |  |  |  |  |  |  |
| Isolates | CZA | CAZ | IPM | AK | CST | AZT | TGC | CIP |
| E. coli DH5α | 0.25 | 0.25 | 0.25 | 1 | 1 | 0.25 | 0.25 | 0.25 |
| E. coli DH5α-KPC-2 | 2 | 128 | 256 | 2 | 2 | >256 | 0.25 | 0.25 |
| E. coli DH5α-KPC-71 | 16 | 128 | 0.5 | 2 | 1 | 8 | 0.5 | 0.25 |
| E. coli TOP10 | 0.25 | 0.25 | 0.25 | 4 | 2 | 0.25 | 0.25 | 0.25 |
| E. coli TOP10-KPC-2 | 4 | 128 | >256 | 4 | 2 | >256 | 0.25 | 0.25 |
| E. coli TOP10-KPC-71 | 32 | 128 | 1 | 4 | 2 | 4 | 0.25 | 0.25 |
| KP13883 | 0.25 | 0.25 | 0.25 | 0.5 | 2 | 0.25 | 1 | 0.25 |
| KP13883-umpA | 0.5 | 0.5 | 0.5 | 1 | 4 | 0.5 | 4 | 0.5 |
| KP13883-△umpA | 0.25 | 0.25 | 0.25 | 0.25 | 1 | 0.25 | 0.25 | 0.25 |
| KP13883-KPC-2 | 2 | 16 | >256 | 0.5 | 8 | >256 | 1 | 0.25 |
| KP13883-(umpA+KPC-2) | 2 | 16 | >256 | 0.5 | 8 | >256 | 8 | 1 |
| KP13883-KPC-71 | 32 | 32 | 8 | 0.5 | 8 | 2 | 1 | 0.25 |
| KP13883-(umpA+KPC-71) | 64 | 128 | 32 | 2 | 16 | 4 | 4 | 2 |
| CRKP4 | 1 | 8 | 32 | 1 | 4 | >256 | 2 | 0.25 |
| CRKP4-umpA | 4 | 64 | 128 | 1 | 4 | >256 | 4 | 1 |
| CRKP4-△umpA | 1 | 8 | 32 | 0.5 | 4 | >256 | 1 | 0.25 |
| CRKP4-KPC-71 | 32 | 256 | 128 | 2 | 8 | >256 | 2 | 0.25 |
| CRKP4-(umpA+KPC-71) | 64 | 256 | >256 | 8 | 16 | >256 | 4 | 1 |
| CRKP8 | 2 | 128 | 256 | 1 | 2 | >256 | 4 | 16 |
| CRKP8-umpA | 4 | 128 | >256 | 2 | 4 | >256 | 8 | 128 |
| CRKP8-△umpA | 1 | 128 | 256 | 0.5 | 1 | >256 | 1 | 16 |
| CRKP8-KPC-71 | 32 | 128 | >256 | 4 | 4 | >256 | 4 | 16 |
| CRKP8-(umpA+KPC-71) | 64 | 128 | >256 | 4 | 4 | >256 | 8 | 128 |
| *^a^* CZA, ceftazidime-avibactam; CAZ, ceftazidime; IPM, imipenem; AK, amikacin; CST, colistin; AZT, aztreonam; TGC, tigecycline; CIP, ciprofloxacin. | | | | | | | | |
|  |  |  |  |  |  |  |  |  |

| TABLE S3 Primers used in this study | | | | |
| --- | --- | --- | --- | --- |
| Experiment | Gene | Primer | Sequence (5'→3') | Product size (bp)R |
| PCR | KPC | KPC-F | ATGTCACTGTATCGCCGT | 913 (2) |
|  |  | KPC-R | GGTGGTGGGCCAATAGAT |  |
|  | ompk35 | ompk35-F | GGATGGAAAGATGCCTTCAG | 1391 (3) |
|  |  | ompk35-R | CATGACGAGGTTCCATTGTG |  |
|  | ompk36 | ompk36-F | GGGAAGAATCGCACGAAATA | 1744 (3) |
|  |  | ompk36-R | TCTTACCAGGGCGACAAGAG |  |
|  | NDM | NDM-F | CCCGGCCACACCAGTGACA | 129 |
|  |  | NDM-R | GTAGTGCTCAGTGTCGGCAT |  |
|  | VIM | VIM-F | TGTCCGTGATGGTGATGAGT | 252 |
|  |  | VIM-R | CCTTCTAGAGAGTGCGTGGG |  |
|  | IMP | IMP-F | GGAATAGAGTGGCTTAATTC | 277 |
|  |  | IMP-R | GCCAAGCTTCTATATTTGCG |  |
|  | OXA | OXA-F | GTCTTTCRAGTACGGCATTA | 721 |
|  |  | OXA-R | GATTTTCTTAGCGGCAACTTA |  |
|  | OXA-23 | OXA-23-F | CCTGATCGGATTGGAGAACCAG | 513 |
|  |  | OXA-23-R | GATGCCGGCATTTCTGACCG |  |
|  | OXA-48 | OXA-48-F | TTGGTGGCATCGATTATCGG | 744 (4) |
|  |  | OXA-48-R | GAGCACTTCTTTTGTGATGGC |  |
|  | CTX-M-1 | CTX-M-1-F | ACGCTGTTGTTAGGAAGTG | 759 |
|  |  | CTX-M-1-R | TTGAGGCTGGGTGAAGT |  |
|  | CTX-M-2 | CTX-M-2-F | ACGCTACCCCTGCTATT | 830 |
|  |  | CTX-M-2-R | CAGAAACCGTGGGTTACGA |  |
|  | CTX-M-9 | CTX-M-9-F | ATGGTGACAAAGAGAGTGCA | 870 (5) |
|  |  | CTX-M-9-R | CCCTTCGGCGATGATTCTC |  |
|  | SHV | SHV-F | TCGGCCTTCACTCAAGGATG | 812 |
|  |  | SHV-R | TCCCGCAGATAAATCACCA |  |
|  | TEM | TEM-F | CATTTCCGTGTCGCCCTTATTC | 800 (4) |
|  |  | TEM-R | CGTTCATCCATAGTTGCCTGAC |  |
|  | ACC | ACC-F | AACAGCCTCAGCAGCCGGTTA | 346 |
|  |  | ACC-R | TTCGCCGCAATCATCCCTAGC |  |
|  | DHA | DHA-F | AACTTTCACAGGTGTGCTGGGT | 405 |
|  |  | DHA-R | CCGTACGCATACTGGCTTTGC |  |
|  | MOX | MOX-F | GCTGCTCAAGGAGCACAGGAT | 520 |
|  |  | MOX-R | CACATTGACATAGGTGTGGTGC |  |
|  | EBC | EBC-F | TCGGTAAAGCCGATGTTGCGG | 302 |
|  |  | EBC-R | CTTCCACTGCGGCTGCCAGTT |  |
|  | FOX | FOX-F | AACATGGGGTATCAGGGAGATG | 190 |
|  |  | FOX-R | CAAAGCGCGTAACCGGATTGG |  |
|  | CIT | CIT-F | TGGCCAGAACTGACAGGCAAA | 462 |
|  |  | CIT-R | TTTCTCCTGAACGTGGCTGGC |  |
| qRT-PCR | qrrsE | q-rrsE-F | TTGACGTTACCCGCAGAAGAA | 71 |
|  |  | q-rrsE-R | GCTTGCACCCTCCGTATTACC |  |
|  | qKPC | q-KPC-F | CGTGACGGAAAGCTTACAAA | 78 |
|  |  | q-KPC-R | AGCCAATCAACAAACTGCTG |  |
|  | qompk35 | q-ompk35-F | CATAGGTGGTATCGTCGCTGCTG | 90 (3) |
|  |  | q-ompk35-R | ACGGCAACAAACTGGACTTCTATGG |  |
|  | qompk36 | q-ompk36-F | GCGAATGCCAGACGAGTCCATG | 126 (3) |
|  |  | q-ompk65-R | TGTAGGCGTGAAAGGCGAAACC |  |
|  | qumpA | q-umpA-F | GTGGGCTTTGTCTTTGCGATGTG | 269 |
|  |  | q-umpA-R | TTGGTGCGTCTGGCGAAGATAATC |  |
|  | qftsI | q-ftsI-F | CCGTGCTCAATACCGTGCCTTATC | 216 |
|  |  | q-ftsI-R | CACTGCGTTCTCCGACCAACC |  |
|  | qglmM | q-glmM-F | TCAAGCAGTTAGGCATCCCGTTTG | 262 |
|  |  | q-glmM-R | CCCTCGGTAAAGCGGACATTCAC |  |
|  | qlpp | q-lpp-F | TACTCTGCTGGCTGGTTGCTC | 108 |
|  |  | q-lpp-R | ATTGCGTTCACGTCGTTGCTC |  |
|  | qpal | q-pal-F | AAAGTGCTGAAAGGGCTGATGATC | 80 |
|  |  | q-pal-R | TCGTTGCTGGCGTTCTTGTTAG |  |
|  | qompA | q-ompA-F | GTGGCGAATAACCTCTGGCAAC | 112 |
|  |  | q-ompA-R | GCAGTGACGACAGAAGAAGATCC |  |
| Cloning expreiment | umpA+arm | umpA+arm-F | TCGAGCTCGGTACCCATGAATAGTGGCTACCTGCATTTTC | 906 |
|  |  | umpA+arm-R | CAAAACAGCCAAGCTTCAAGAATTCTGTTGCTGCGGAC |  |
|  | KPC+WP | KPC+WP-F | GTCTTGACATATAGGTTAATGTCATG | 1182 |
|  |  | KPC+WP-R | TTTTCAGAGCCTTACTGCCCG |  |
|  | umpA-U1 | umpA-U1-F | GGCGGCCTTTATGGAGCGT | 300 |
|  |  | umpA-U1-R | CTGTCACCATAAAGACTTGTTATTC |  |
|  | umpA-D1 | umpA-D1-F | GGAACCATGAAACAGTATCTTGATT | 300 |
|  |  | umpA-D1-R | CCACTGTTTGCCGTAGACCG |  |
|  | umpA-U2 | umpA-U2-F | ACATGATTACGAATTGGCGGCCTTTATGGAGCG | 335 |
|  |  | umpA-U2-R | CACTGATTAAGCATTGGTAACTGTCACCATAAAGACTTGT |  |
|  | umpA-D2 | umpA-D2-F | TAAACAAATAGGGGTTCCGCGGGAACCATGAAACAGTA | 336 |
|  |  | umpA-D2-R | GGCCAGTGCCAAGCTCCACTGTTTGCCGTAGACCG |  |
|  | Amp | Amp-F | GTCTTTATGGTGACAGTTACCAATGCTTAATCAGTGAGG | 1000 |
|  |  | Amp-F | ATACTGTTTCATGGTTCCCGCGGAACCCCTATTTGTTTA |  |
| R, Reference. |  |  |  |  |

**References**

1. Jousset AB, Oueslati S, Emeraud C, Bonnin RA, Dortet L, Iorga BI, Naas T. 2021. KPC-39-Mediated Resistance to Ceftazidime-Avibactam in a Klebsiella pneumoniae ST307 Clinical Isolate. Antimicrob Agents Chemother 65:e0116021.

2. Guo Y, Liu N, Lin Z, Ba X, Zhuo C, Li F, Wang J, Li Y, Yao L, Liu B, Xiao S, Jiang Y, Zhuo C. 2021. Mutations in porin LamB contribute to ceftazidime-avibactam resistance in KPC-producing Klebsiella pneumoniae. Emerg Microbes Infect 10:2042-2051.

3. Clancy CJ, Chen L, Hong JH, Cheng S, Hao B, Shields RK, Farrell AN, Doi Y, Zhao Y, Perlin DS, Kreiswirth BN, Nguyen MH. 2013. Mutations of the ompK36 porin gene and promoter impact responses of sequence type 258, KPC-2-producing Klebsiella pneumoniae strains to doripenem and doripenem-colistin. Antimicrob Agents Chemother 57:5258-65.

4. Zeng W, Liao W, Zhao Y, Wang L, Shu H, Jia H, Chen T, Zhang Y, Zhou T, Wu Q. 2022. A Selective Medium for Screening Ceftazidime/Avibactam Resistance in Carbapenem-Resistant Enterobacterales. Front Microbiol 13:956044.

5. Eckert C, Gautier V, Saladin-Allard M, Hidri N, Verdet C, Ould-Hocine Z, Barnaud G, Delisle F, Rossier A, Lambert T, Philippon A, Arlet G. 2004. Dissemination of CTX-M-type beta-lactamases among clinical isolates of Enterobacteriaceae in Paris, France. Antimicrob Agents Chemother 48:1249-55.
