# Supplementary figures and images for: Tandem amplification of the umpA allele contributes to ceftazidime-avibactam heteroresistance in clinical carbapenem-resistant Klebsiella pneumoniae isolates
Source: Microbiol Spectr. 2026 Apr 13;14(5):e01722-25. doi: 10.1128/spectrum.01722-25 (PMC13141876; doi:10.1128/spectrum.01722-25)

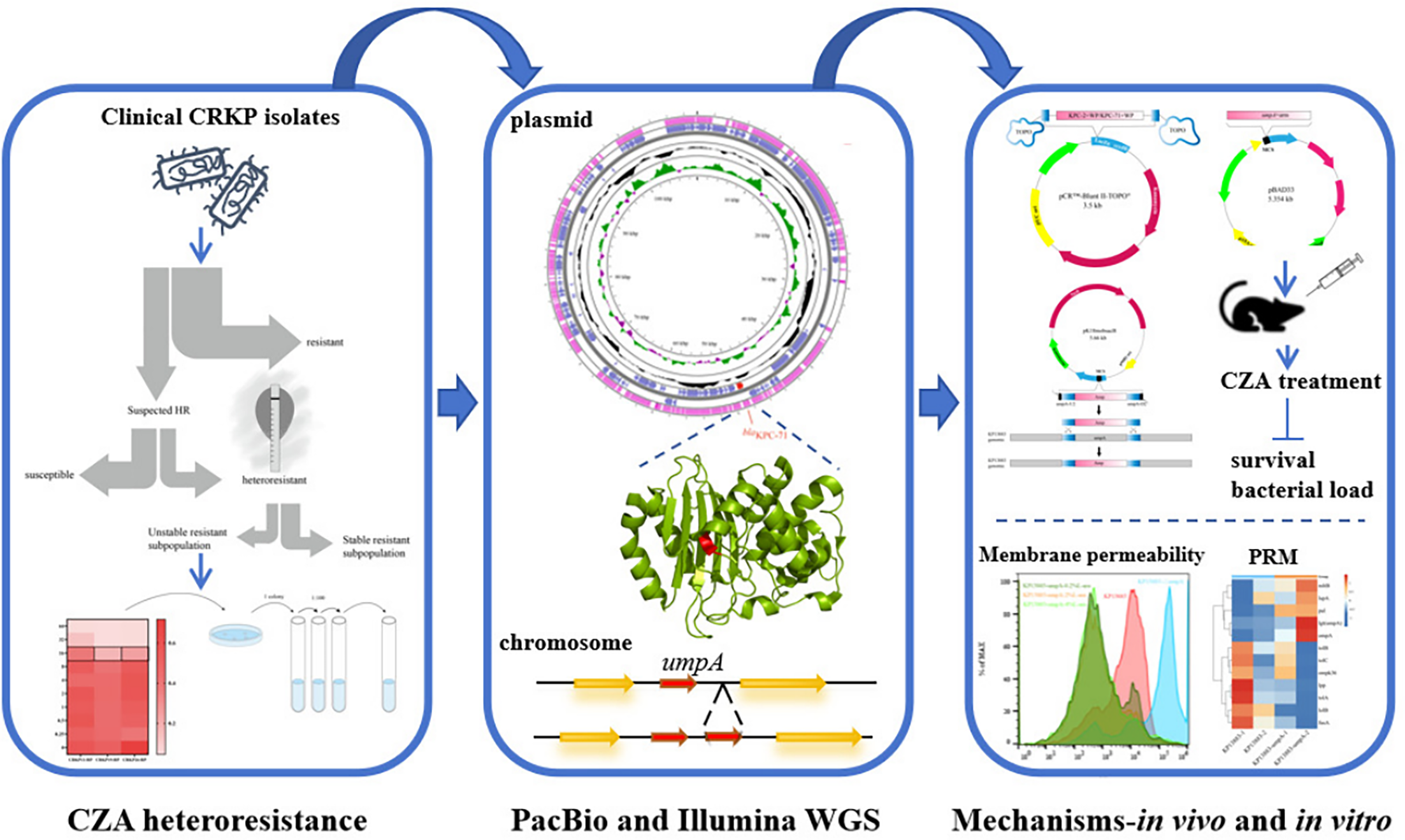

Supplement: Graphical abstract — Mechanisms of CZA heteroresistance in clinical CRKP isolates. [file spectrum.01722-25-s0002.tif]
